# Effects of BioCell Collagen® on connective tissue protection and functional recovery from exercise in healthy adults: a pilot study

**DOI:** 10.1186/1550-2783-11-S1-P48

**Published:** 2014-12-01

**Authors:** Hector L Lopez, SM Habowski, JE Sandrock, AW Kedia, TN Ziegenfuss

**Affiliations:** 1 The Center for Applied Health Sciences, Stow, Ohio, USA

## Background

The extracellular matrix (ECM) of muscle, tendon, and ligament is sensitive to exercise-induced mechanical stimuli. Exercise-induced muscle damage is associated with not only myofibrillar injury, but also the involvement of connective tissue elements such as collagen, proteoglycans (PG), tendon and ligament. However, little is known about the impact of nutritional agents and metabolic optimization for enhancing adaptation and recovery of the connective tissue elements that support musculoskeletal function. BioCell Collagen® (BCC) is a patented hydrolyzed chicken sternal cartilage extract that contains a naturally-occurring matrix of hydrolyzed collagen type II, and low molecular weight glycosaminoglycans such as chondroitin sulfate and hyaluronic acid. The purpose of this pilot study was to determine the potential impact of daily supplementation with BCC on functional indices and molecular biomarkers of recovery from intense exercise, and identify effect sizes on various outcome measures.

## Methods

Eight healthy, recreationally active subjects (29.3 ± 9.2 y, 173.1 ± 8.2 cm, 77.3 ± 13.5 kg) volunteered to participate in this study and were randomized in a double-blind, placebo-controlled fashion to ingest either 3 g of placebo or BioCell Collagen® daily over a 6-week period prior to an upper body muscle-damaging resistance exercise challenge (UBC) on day 43, and a re-challenge on day 46. At the end of the 6-week supplementation period, participants completed a UBC consisting of 8 sets of barbell bench press at 75% of body weight load to exhaustion with a 4/0/X repetition tempo and 90 seconds rest between sets; the UBC exercise challenge was repeated 72 hours later to assess recovery of function. Consent to publish the results was obtained from all participants.

## Results

Daily intake of BCC for 6-weeks attenuated an increase in serum markers for muscle tissue damage in response to bench press exercise, creatine kinase (CK), lactate dehydrogenase (LDH), and C-reactive protein (CRP). Change in CK: +20 U/L (BCC) vs. +4726 U/L (placebo); change in LDH: -3.5 U/L (BCC) vs. +82.9 U/L (placebo); change in CRP: +0.07 mg/L (BCC) vs. +0.7 mg/L (placebo). In terms of performance, the decrement in bench press repetitions to failure was only 49% (day 43) and 43% (day 46) in the BCC group vs. 60% (day 43) and 55% (day 46) in the placebo group.

## Conclusion

The preliminary data of this proof-of-concept study suggests that daily intake of BCC for 6 weeks may favorably impact key biochemical markers of connective and skeletal muscle tissue damage and enhance stress resilience following intense resistance exercise. Supplementation was well tolerated and did not adversely affect markers of health or side effect profiles.

